# CircSMC3 regulates gastric cancer tumorigenesis by targeting miR‐4720‐3p/*TJP1* axis

**DOI:** 10.1002/cam4.3057

**Published:** 2020-04-20

**Authors:** Tianfang Xia, Zhenguo Pan, Jie Zhang

**Affiliations:** ^1^ Department of General Surgery The Affiliated Huaian No.1 People’s Hospital of Nanjing Medical University Huaian City Huaiyin District Jiangsu Province China; ^2^ Department of Gastroenterology The Affiliated Huaian No.1 People’s Hospital of Nanjing Medical University Huaian City Jiangsu Province China

**Keywords:** circRNA, circSMC3, gastric cancer, miR‐4720‐3p, *TJP1*

## Abstract

Circular RNAs (circRNAs) are identified to play an evident role in many human cancers, such as gastric cancer. However, the potential mechanisms underlying the circRNA‐induced pathogenesis in gastric cancers are still elusive. The present study is designed to unfold the mechanism by which circRNAs involve in gastric cancer progression. Using circRNAs microarray, we detected the dysregulated circRNAs and identified an upregulated circRNA, circSMC3 (hsa_circ_0000260), in gastric cancer tissues. Patients with high circSMC3 expression levels had a poor overall survival via Kaplan‐Meier survival analysis implied that gastric cancer. Functionally, loss of circSMC3 abolished the proliferation and motility of gastric cancer cells. Mechanically, circSMC3 decreased miR‐4720‐3p expression by acting as a miRNA sponge, and tight junction protein 1 (*TJP1*) 3′UTR was identified to be the target of miR‐4720‐3p, contributing to a circSMC3/miR‐4720‐3p/*TJP1* axis. Thus, our results indicate that circSMC3 promotes gastric cancer cell proliferation and motility through miR‐4720‐3p/*TJP1*.

## Graphical abstract

CircSMC3 acted as a sponge for miR‐4720‐3p and decreased its expression, and tight junction protein 1 (*TJP1*) 3′UTR was found to be the target of miR‐4720‐3p, contributing to a circSMC3/miR‐4720‐3p/*TJP1* axis.

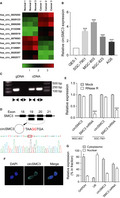

## 1 INTRODUCTION

Gastric cancer, a most common malignant tumor worldwide, is characterized by its high incidence and mortality.^1^ Tumor metastasis is a common and major obstacle to increase the survival of patients with gastric cancer.^2^ Although many advances had been achieved in surgery, chemotherapy, and molecular targeted therapies, the morbidity and mortality of gastric cancer are still not optimistic.^3^, ^4^ The challenge of treating gastric cancer includes not only tumor recurrence or metastasis, but also uncertain and nonspecific therapeutic targets.^3^, ^5^ We need a better knowledge of gastric cancer pathogenesis for advancing and improving available therapeutic targets and markers.^6^

Circular RNA (circRNA) is a new subtype of noncoding RNA; they are covalently closed loop RNAs formed by 3′ end to 5′ end joining RNA fragments.^7^ Despite circRNAs had been identified for more than 40 years, they had only received the researchers’ attention in recent years.^8^ Using high‐throughput sequencing method, more than 30 000 circRNAs had been identified. Including gastric tissues, circRNAs are ubiquitously expressed in many tissues.^9^, ^10^, ^11^ Some circRNAs had been investigated in gastric cancer. For instance, circular RNA_LARP4 negatively correlated with miR‐424 expression; circLARP4/miR‐424 axis targeted* LATS1* to regulate gastric cancer cell proliferation and invasion^12^; the downregulated circRNA_100269 suppressed tumor cell proliferation and growth by interacting with miR‐630^13^; and circPVT1 served as a prognostic marker and proliferative factor in gastric cancer.^14^ These phenomena indicated that circRNAs played a significant role in the different progresses of gastric cancer. However, more circRNAs need to be explored in gastric cancer.

In the present study, we detected a novel circRNA (hsa_circ_0000260), named circSMC3, which is generated from SMC3 gene locus with spliced length 305 nt. We revealed that circSMC3 was elevated in both gastric cancer tissue and cells. Gastric cancer cell growth and motility were inhibited when we knocked down circSMC3. Mechanically, circSMC3 served as a miRNA sponge to decrease miR‐4720‐3p. *TJP1*’s 3′UTR was identified as the target of miR‐4720‐3p. The levels of circSMC3, miR‐4720‐3p, and *TJP1* in tissues were examined by RT‐qPCR or immunohistochemistry (IHC). We found a negative correlation between circSMC3 and miR‐4720‐3p levels. Furthermore, miR‐4720‐3p and *TJP1* also affected gastric cancer cell growth and motility. Taken together, our results indicated that circSMC3/miR‐4720‐3p/*TJP1* axis gave us a new direction for the potential treatment of gastric cancer.

## 2 MATERIALS AND METHODS

### 2.1 Clinical specimens

The paired gastric cancer tissues and adjacent normal tissues were collected from the patients in The Affiliated Huaian No.1 People's Hospital of Nanjing Medical University from 2015 to 2019. The specimens were taken after tumor excision within less than 10 minutes, then we stored the specimens at −80°C immediately till application in the experiments. This study was approved by The Ethics Committee of The Affiliated Huaian No.1 People's Hospital of Nanjing Medical University (2017210017), and we had obtained written informed consents from all gastric cancer patients.

### 2.2 Cell culture

All cells were bought from American type culture collection (ATCC) or The Cell Bank of Type Culture Collection of Chinese Academy of Sciences. Short tandem repeat (STR) DNA profiling method was used to authenticate all cell lines in December 2017. Human gastric cell lines, for instance AGS, GES‐1, MGC‐803, BGC‐823, and SGC‐7901 were incubated in DMEM (Gibco), which was mixed with 10% fetal bovine serum (FBS) (Gibco), and 1% penicillin/streptomycin (Invitrogen, USA). Cells were cultured in an atmosphere at 37°C with 5% CO_2_.

### 2.3 Quantitative real‐time PCR (RT‐qPCR)

According to the manufacturers’ instructions, total RNA was obtained from cells or tissues by TRIzol reagent (Invitrogen). The concentration of RNA was determined by NanoDrop ND2000 (Thermo Scientific.) to examine the purity and quantify of extracted total RNA. HiScript II Q RT SuperMix for qPCR Kit (Vazyme Biotech Co., Ltd) was used for total RNA reverse transcription. Primers used for RT‐qPCR were synthesized by Tsingke Biological Technology (Nanjing). ChamQ SYBR qPCR Master Mix (without ROX) (Vazyme Biotech Co., Ltd) was used for RT‐qPCR according to the manufacturer's instructions, in a Roche LC 96 qPCR system (Roche). The PCR reaction program started at 95°C for 2 minutes, 40 cycles for 95°C for 10 seconds followed by 60°C for 30 seconds. *GAPDH* or U6 was the internal reference of measuring qPCR results. Target gene relative levels were measured by 2^−ΔΔCT^ method. The RT‐qPCR primers of circSMC3 were Forward: 5'‐CAGATCGAGACCCAGCAAA‐3'; Reverse: 5'‐GCAGGTTTTCATTGAGCTTT‐3'.

### 2.4 Cell treatment

Lipofectamine 2000 (Life Technologies) was used for plasmid or small interfering RNA (siRNA) transfection. Lentiviral expression systems (psPAX2, pMD2.G, and sh‐circSMC3) were generated to transduce gastric cancer cells. The siRNAs were designed for transient knockdown circSMC3, and they were synthesized by GenePharma Co., Ltd.. The siRNA sequence crossing the circSMC3 junction site is: 5′‐CAUGCCUAAGGUGACCAAGU‐3′.

### 2.5 RNA isolation of nuclear and cytoplasmic fractions

According to the manufacturer's instructions, we employed the NE‐PER^™^ Nuclear and Cytoplasmic Extraction Reagents Kit (Thermo Scientific) to isolate and collect cytosolic and nuclear fractions. The expression levels of *GAPDH* (cytoplasmic control transcript) and U6 (nuclear control transcript) were examined in nuclear and cytoplasmic fractions using RT‐qPCR.

### 2.6 Cell counting kit‐8 (CCK‐8)

1 × 10^3^ gastric cancer cells were seeded into a 96‐well plate. 450‐nm absorbance was measured after incubating the cells with 100‐µL CCK‐8 kit (Dojindo Laboratories) for 1 hour.

### 2.7 Plate clone formation

We digested gastric cancer cells and plated 1 × 10^4^ cells on a 6‐well plate. Then the cells were cultured in a CO_2_ incubator at 37°C for 2 weeks until the cells had formed colonies. Cells were washed twice with 2‐mL phosphate buffer saline (PBS). The cells were then fixed by 4% paraformaldehyde (PFA) for 15 minutes at room temperature. Next, the cell colonies were stained at room temperature by 0.5% crystal violet for 20 minutes. The cells were photographed after washing three times with PBS.

### 2.8 Fluorescent in situ hybridization (FISH)

According to the manufacturer's instructions, the signals of circSMC3 were detected by Fluorescent In Situ Hybridization Kit (RiboBio). Briefly, 1 × 10^3^ SGC‐7901 cells were plated into coverslips, then the cells were fixed by 4% PFA. Next, the cell membrane was permeabilized at room temperature by 0.5% Triton X‐100 in DEPC‐PBS for 15 minutes, then subjected to three 10‐min washes with 2 × saline sodium citrate (SSC) buffer. The cells were incubated with the hybridization buffer containing a 5′‐Cy3‐labeled circSMC3 probe (GenScript) overnight at 37°C. The following day, we washed the coverslips three times with 2 × SSC at room temperature. The samples were then incubated at room temperature for 20 minutes with 4',6‐diamidino‐2‐phenylindole (DAPI). The stained cells were photographed via Zeiss Axiovert 200M laser scanning confocal microscope (Carl Zeiss). The circSMC3 probe sequence containing back‐splice junction site was 5′‐CGAUGGCUGACUUGGUCACCUUAGGCAUGAAGGUUUUC‐3′.

### 2.9 Animal experiment

The mice care and whole experimental protocols were approved by The Experimental Animal Welfare Ethics Committee of The Affiliated Huaian No.1 People's Hospital of Nanjing Medical University (2017310524). Animal experiments were performed in compliance with the guidelines of the Animal Research Ethics Board of MMM. We purchased 4 weeks old male BALB/c nude mice from the Charles River laboratories and the mice were maintained under pathogen‐free conditions during 2018‐2019. In the back flank, mice (five in each group) were subcutaneously injected 1 × 10^7^ cells in 200‐µL cell suspension. The tumors were measured every week after the tumor was visible and the volume of the tumors were calculated following the formula: volume (1/2 × length × width^2^).

### 2.10 Dual‐Luciferase reporter assay

CircSMC3 segment (100 bp) or *TJP1* 3′UTR was constructed into pGL3‐control plasmid. Either target sequence or wild‐type seed region was co‐transfected into HEK‐293T cells that were cultured in 48‐well plates with 50‐ng Renilla luciferase reporter plasmid using Lipofectamine 2000. The Dual‐Luciferase^®^ Reporter Assay System (Promega) was used to measure the luciferase activities after 48‐hours transfection.

### 2.11 Western blot assay

Proteins were extracted from cells or tissues using detergent‐containing RIPA lysis buffer. Equal amounts of total proteins were subjected to sulfate‐polyacrylamide gel electrophoresis (SDS‐PAGE) and proteins were transferred to 0.45‐μm polyvinylidene difluoride (PVDF) membrane (Millipore). The PVDF membrane was incubated with primary antibodies as follows: anti‐TJP1 (Abcam) and anti‐GAPDH (Santa Cruz) after blocking with 5% nonfat milk. Proteins were visualized through horseradish peroxidase (HRP) conjugated secondary antibody and peroxide LumiGLO reagent system (Cell Signaling Technology).

### 2.12 RNA‐RNA pull‐down assay

RNA‐RNA pull‐down assay was employed to detect potential binding between circSMC3 and miR‐4720‐3p. The biotin‐labeled RNA probe targeting circSMC3 was generated from GenScript Biotech Co., Ltd.. The probe sequence was 5′‐AAAACCUUCAUGCCUAAGGUGACCAAGUCAGCCAUCGG‐3′. The probe was mixed with the lysate of gastric cancer cells for 3 hours at 4°C. Thereafter, the complexes were mixed with streptavidin magnetic beads (Thermo Fisher Scientific) for 2 hours. At last, the RNA was eluted and the level of circSMC3‐bound miR‐4720‐3p was examined by RT‐qPCR.

### 2.13 Statistical analysis

Mean ± SD values were expressed for the values. All statistical results were analyzed using PRISM 8.0.1 software. Significances were analyzed by unpaired Student's *t* test. *P* values < .05 were considered statistically significant.

## 3 RESULTS

### 3.1 circSMC3 is increased in gastric cancer tissues

**Figure 1:**
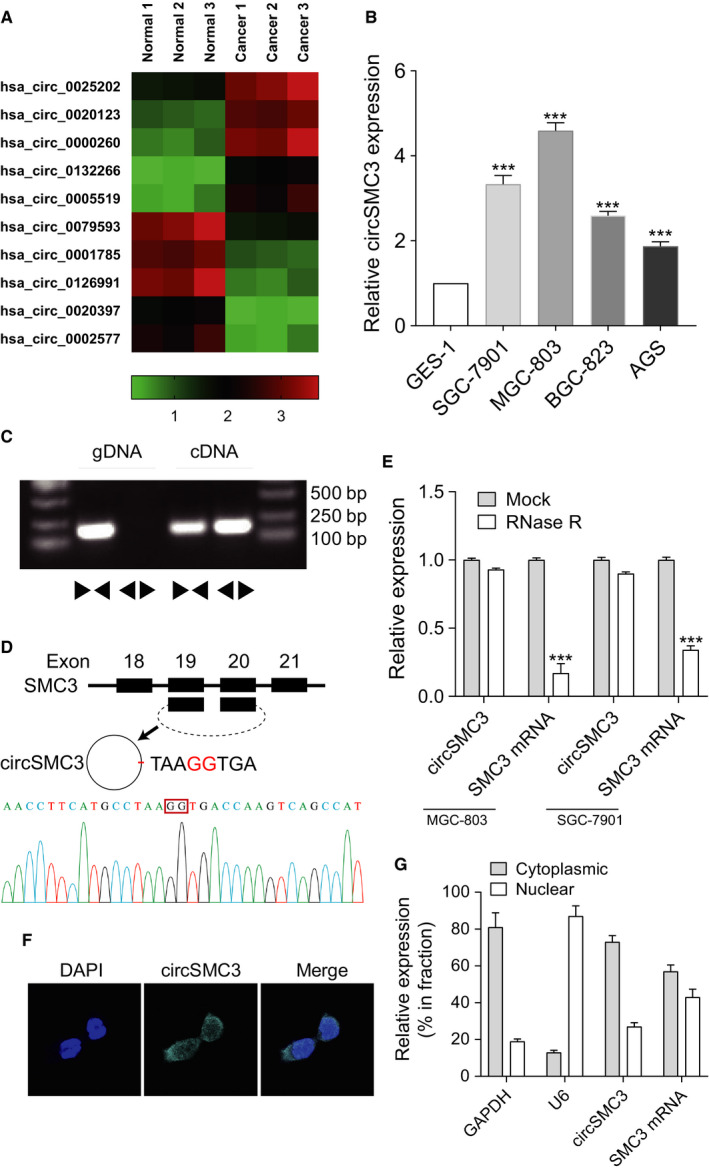
circSMC3 is overexpressed in the gastric cancer tissue and cells. A, The heatmap showed the representative dysregulated circRNAs in gastric cancer tissues. B, circSMC3 (hsa_circ_0000260) was upregulated in the different gastric cancer cells. C, We detected the expression levels of the back‐spliced and canonical forms of SMC3 in cDNA and gDNA from gastric cancer cells by PCR and an agarose gel electrophoresis assay. D, Upper panel: Schematic representation of circSMC3 formation. Lower panel: The back‐splice junction site of circSMC3 was validated by RT‐PCR followed by Sanger sequencing. E, RT‐qPCR for the abundance of circSMC3 and SMC3 mRNA in gastric cancer cells treated with RNase R. F, We found that circSMC3 was most localized in the cytoplasm by FISH analysis. G, circSMC3 was enriched in MGC‐803 cytoplasm fraction. Levels of circSMC3,* SMC3* mRNA, *GAPDH*, and U6 RNA in purified MGC‐803 nuclear and cytoplasmic fractions were detected by RT‐qPCR. Notes: ***Indicates a significant difference (*P* < .001) calculated by Student's *t* test

To detect abnormally expressed circRNAs in gastric cancer tissues, three gastric cancer tissues and paired adjacent normal tissues were subjected to ArrayStar circRNA microarray. Among the deregulated circRNAs, we exhibited five circRNAs that were upregulated or downregulated in Figure 1A. In other types of cancer, some of these circRNAs had previously been studied. However, there are no reports on circSMC3 (hsa_circ_0000260) in the literature. Using RT‐qPCR, we detected the expression of circSMC3 in gastric cancer or normal cells. To compare the circSMC3 levels between normal cells and the established cell lines of gastric cancer, the circSMC3 levels were examined in GES‐1, AGS, SGC‐7901, BGC‐823, and MGC‐803 cells. The levels of circSMC3 in gastric cancer cells were significantly higher than that in normal cells (Figure 1B). Using genomic DNA (gDNA) or cDNA as templates from SGC‐7901 cell lines, the circSMC3 amplification products were only detected in cDNA but not in gDNA by divergent primers (Figure 1C). The junction site of circSMC3 was validated by Sanger sequencing followed by RT‐PCR (Figure 1D). With divergent and convergent primers, we performed RT‐qPCR assay and found that circSMC3, but not linear *SMC3* or *GAPDH*, could resist RNase R digestion, an enzyme that has no digestion on circRNAs (Figure 1E). Additionally, the FISH experiment showed that SGC‐7901 exhibited a dominantly cytoplasmic distribution of circSMC3 (Figure 1F). We isolated nuclear and cytoplasmic fractions from MGC‐803 cells. The isolated cytoplasmic fraction showed a higher level of circSMC3 than the nuclear fraction (Figure 1G).

**Figure 2:**
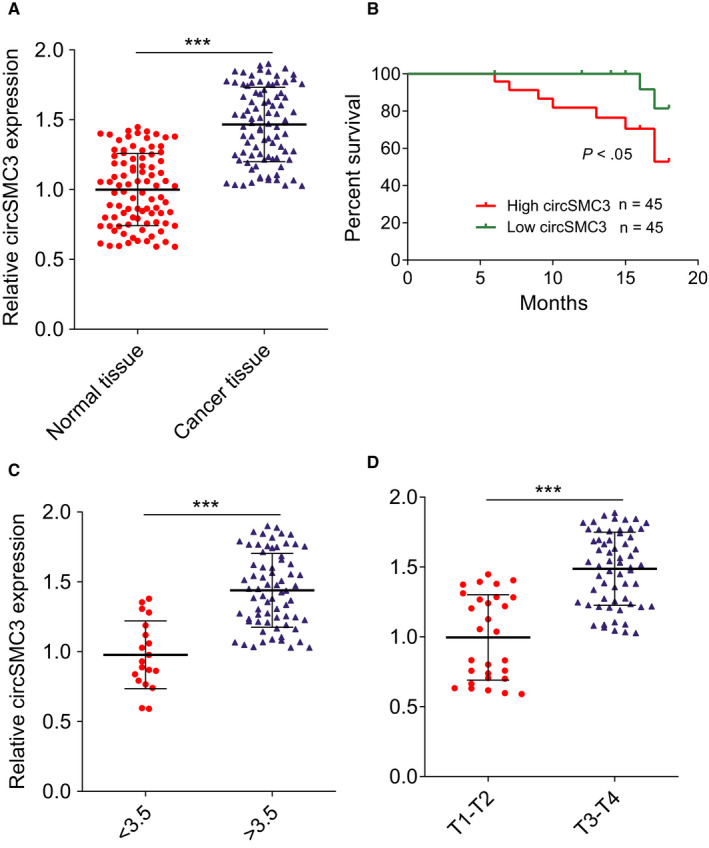
circSMC3 negatively correlates with patient prognosis. A, RT‐qPCR analysis was carried out to detect the expression level of circSMC3 in 90 gastric cancer tissues and paired noncancerous tissues. B, Kaplan‐Meier univariate analysis of overall survival in gastric cancer patients with high (above median) versus low (below median) circSMC3 levels was shown; *P* < .05 [log‐rank test]. C, The circSMC3 was examined in gastric cancer tissues with < 3.5 cm (n = 19) and > 3.5 cm (n = 71). D, The circSMC3 was examined in gastric cancer tissues in T1‐T2 stage (n = 29) and T3‐T4 stage (n = 61). Notes: ***Indicates a significant difference (*P* < .001) calculated by Student's *t* test

Next, we detected the circSMC3 levels in 90 paired gastric cancer tissues and normal tissues. The results indicated that circSMC3 was significantly increased in gastric cancer tissues compared to the normal tissues (Figure 2A). The correlation between clinicopathological features of gastric cancer patients and circSMC3 levels was analyzed. Briefly, using median expression values, we divided 90 patients into two groups, the high and low expression groups, depending on the fold change (2^‐ΔΔCT^). The results revealed that the significant high levels of circSMC3 in patients were correlated with tumor size and advanced T classification (Table 1). Additionally, the overall survival information was followed‐up from the patients previously and then analyzed using the Kaplan‐Meier method. It showed that patients who had high levels of circSMC3 within their gastric cancer tissues had significant shorter overall survival (Figure 2B). Additionally, circSMC3 was upregulated in gastric cancer tissues that are larger than 3.5 cm (Figure 2C), and also was increased in the group of gastric cancer tissues in advanced stages (Figure 2D), implying the positive association between gastric cancer progression/metastasis and circSMC3 expression.

**Table 1:** Correlation between circSMC3 expression and clinicopathologic characteristics of gastric cancer patients

Characteristics	circSMC3			
	Cases	High	Low	*P* value
Gender				
Male	41	21	20	.32
Female	49	20	29	
Age(years)				
<60	17	8	9	.18
≥60	73	47	26	
Tumor size(cm)				
<3.5	19	11	8	.03
≥3.5	71	58	13	
Histological grade				
Well‐moderately	26	11	15	.99
Poorly signet	64	27	37	
Clinical stage				
II	43	17	26	.37
III	47	23	24	
T classification				
T1‐T2	29	12	17	.03
T3‐T4	61	40	21	
N classification				
T1‐T2	22	12	10	.39
T3‐T4	68	30	38	

### 3.2 circSMC3 knockdown represses cell growth and motility

**Figure 3:**
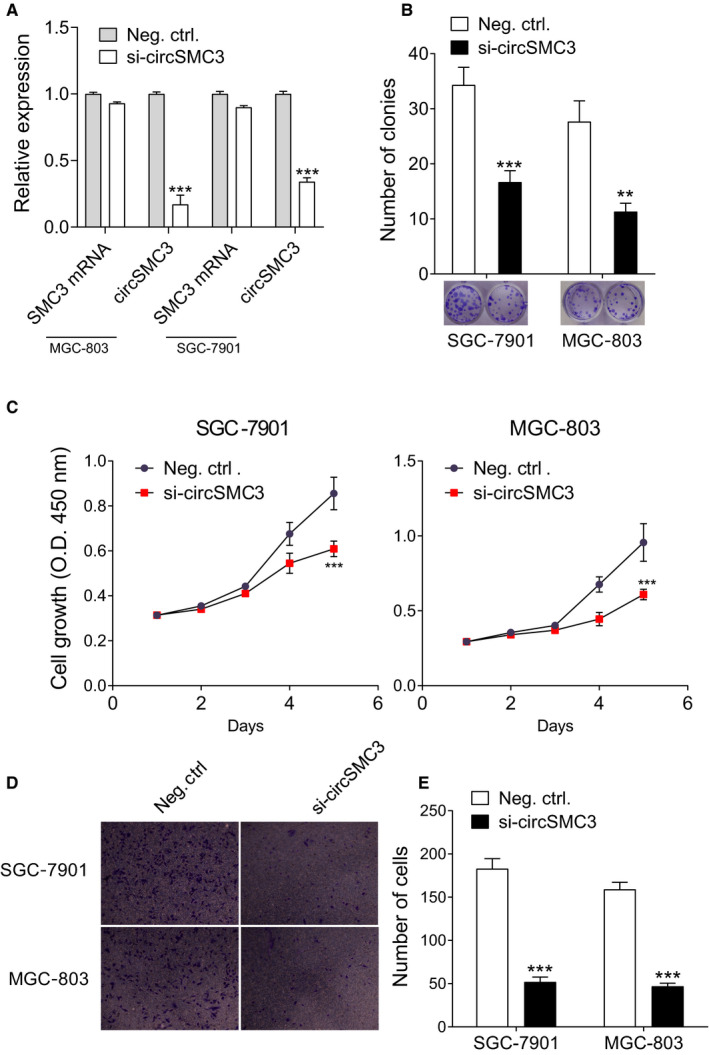
circSMC3 knockdown represses the proliferation and motility of gastric cancer cells. A, Transfection efficiency of circSMC3 into GC cells (MGC‐803 and SGC‐7901) was examined by qPCR. B, Clone formation assay demonstrated the clone number in the circSMC3 knockdown transfection group and the control transfection. C, CCK‐8 assay showed the inhibition of circSMC3 knockdown on the cell proliferation ability. D, The representative images of transwell assay after knocking down circSMC3. E, The statistical results of D. Notes: **Indicates a significant difference (*P* < .01); ***indicates a significant difference (*P* < .001) calculated by Student's *t* test

To investigate the role of circSMC3 in gastric cancer cells, we used siRNA that targets at the circSMC3 junction site to knockdown circSMC3 in gastric cancer cells (MGC‐803 and SGC‐7901) (Figure 3A). The results of clone formation assay showed a reduced number of clones in the circSMC3 knockdown transfection group (Figure 3B). Next, CCK‐8 assay was carried out to evaluate the effect of circSMC3 knockdown on cell proliferation. Gastric cancer cells transfected with circSMC3 siRNAs had an inhibitory effect on cell growth (Figure 3C). We performed transwell invasion assay to determine whether circSMC3 regulated the motility of gastric cancer cells. As shown in Figure 3D,E, the invasion of gastric cancer cells was significantly inhibited by circSMC3 siRNAs. These results suggested that circSMC3 knockdown repressed the proliferation and motility of gastric cancer cells.

### 3.3 circSMC3 targets miR‐4720‐3p as a miRNA sponge

**Figure 4:**
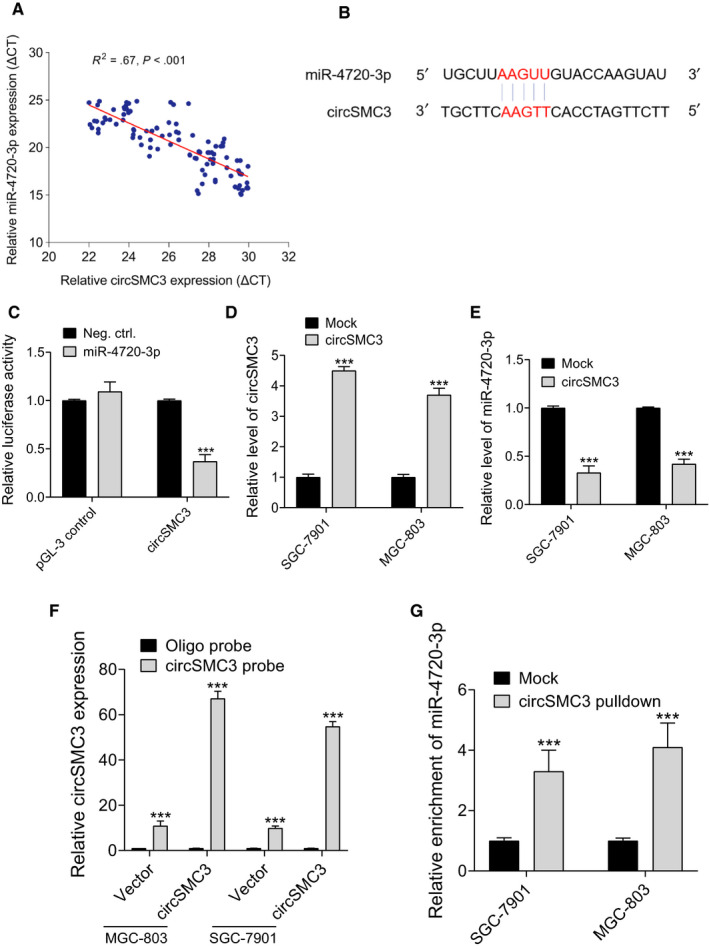
circSMC3 targets miR‐4720‐3p as a miRNA sponge. A, The correlation analysis of expression of circSMC3 and miR‐4720‐3p in gastric cancer tissues. B, miR‐4720‐3p had the complementary sites with the circSMC3. C, The luciferase activity on behalf of the molecular binding of circSMC3 and miR‐4720‐3p was tested. D, circSMC3 levels in the overexpressing gastric cancer cells were tested using RT‐PCR. E, circSMC3 negatively regulated the expression of miR‐4720‐3p in gastric cancer cells. F, Lysates from MGC‐803 and SGC‐7901 cells with circSMC3 vector were subjected to biotinylation‐cirSMC3 pull‐down assay, and expression levels of circSMC3 were measured by RT‐qPCR. G, miR‐4720‐3p were measured by RT‐qPCR in F. Notes: ***Indicates a significant difference (*P* < .001) calculated by Student's *t* test

To elucidate circRNA‐miRNA interaction potentials, bioinformatics methods (circbank, CircInteractome, TargetScan, and Miranda) were used to predict potential binding sites of miRNAs in circSMC3.^15^, ^16^ The results of this study found that miR‐4720‐3p may be the target of circSMC3. Then, we examined the expression correlations between circSMC3 and miR‐4720‐3p, and the results indicated that circSMC3 was negatively correlated with miR‐4720‐3p (Figure 4A). The miR‐4720‐3p complementary binding site to circSMC3 is shown in Figure 4B. The luciferase activity assay confirmed the molecular interaction between circSMC3 and miR‐4720‐3p (Figure 4C). Next, we overexpressed circSMC3 in MGC‐803 and SGC‐7901 cells (Figure 4D) and found that circSMC3 could reduce the expression of miR‐4720‐3p (Figure 4E). To detect the circSMC3/miR‐4720‐3p interaction, RNA‐RNA pull‐down assay was carried out and we found that miR‐4720‐3p was highly enriched by circSMC3 pull‐down (Figure 4F,G). Together, these results revealed that circSMC3 serves as a miRNA sponge for miR‐4720‐3p.

### 3.4 *TJP1* is the target of circSMC3/miR‐4720‐3p

**Figure 5:**
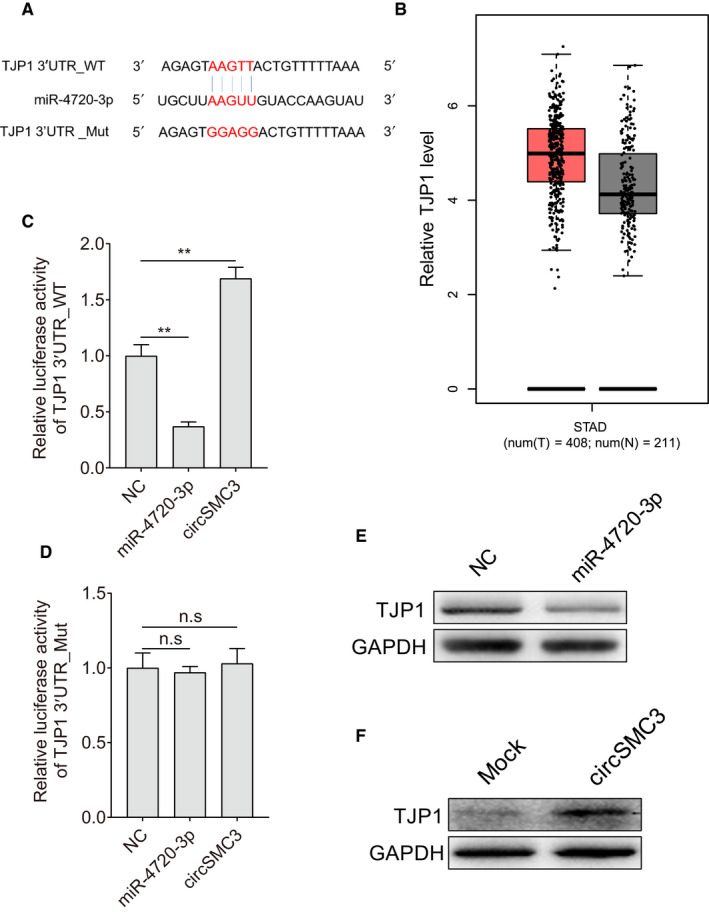
*TJP1* is the target of miR‐4720‐3p. A, Diagram of 3′UTR of *TJP1* containing wild‐type and mutation binding sites of miR‐4720‐3p was shown. B, The expression of *TJP1* in the gastric cancer (stomach adenocarcinoma) based on the GEPIA database (http://gepia.cancer‐pku.cn) was shown. C, miR‐4720‐3p mimics decreased luciferase activities in the cells transfected with plasmids containing wild‐type 3′UTR of *TJP1*, while circSMC3 increased luciferase activities in that. D, miR‐4720‐3p and circSMC3 had no effect on the luciferase activity of *TJP1* 3′UTR_Mut. E, miR‐4720‐3p mimics decreased protein levels of *TJP1*. F, circSMC3 increased protein levels of* TJP1*. Notes: **Indicates a significant difference (*P* < .01) calculated by Student's *t* test

Further experiments were carried out to identify the downstream target of circSMC3 and miR‐4720‐3p. Bioinformatics analysis (Pita, Miranda, TargetScan, Findtar, and RNAhybrid) was then performed to predict the putative miR‐4720‐3p targets. The *TJP1* mRNA 3′UTR was predicted to have complementary sites with miR‐4720‐3p (Figure 5A). Furthermore, the levels of *TJP1* in gastric cancer tissues were elevated according to the GEPIA database (http://gepia.cancer‐pku.cn) (Figure 5B). Then, luciferase reporter assay showed that miR‐4720‐3p could inhibit luciferase activity of the *TJP1* 3′UTR reporter (Figure 5C), but has no effect on *TJP1* 3′UTR_mutant reporter (Figure 5D). Western blot analysis indicated that the levels of *TJP1* were inhibited after transfecting miR‐4720‐3p (Figure 5E). Furthermore, western blot illustrated that *TJP1* was elevated after transfecting circSMC3 (Figure 5F).

### 3.5 circSMC3/miR‐4720‐3p regulates *TJP1* in vivo

**Figure 6:**
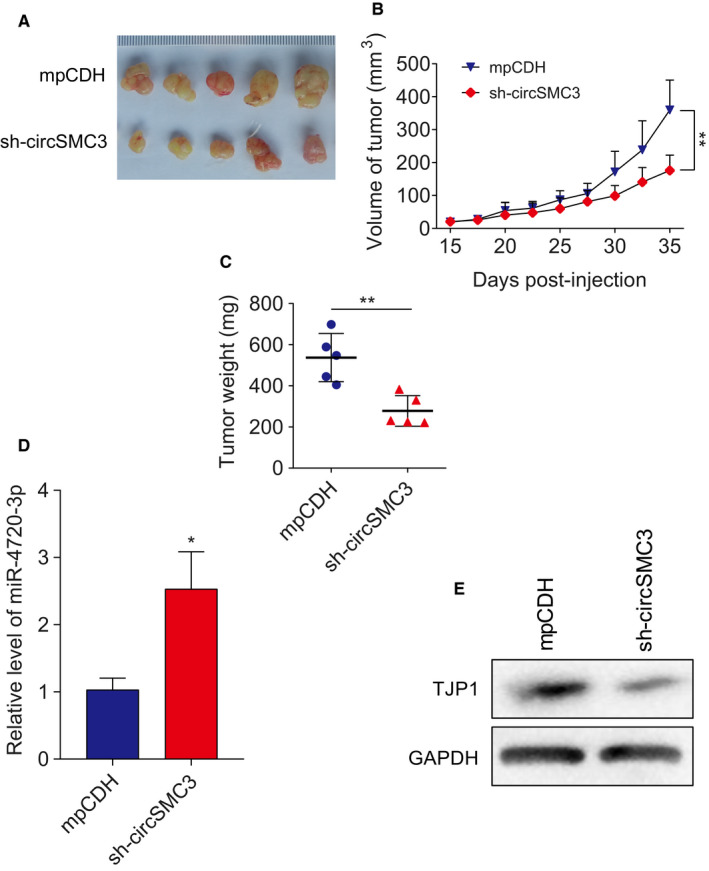
circSMC3/miR‐4720‐3p regulates *TJP1* in vivo. A, Excised tumors from circSMC3 knockdown group (sh‐circSMC3) were much smaller than the negative control group. B, Tumor growth curves of the excised tumors were shown. C,Tumor weight of the excised tumors was shown. D, Expression of miR‐4720‐3p was much higher in sh‐circSMC3 tumor group than in control group measured by RT‐qPCR. E, Expression of TJP1 was decreased in sh‐circSMC3 group than in control group measured by western blot. Notes: *Indicates a significant difference (*P* < .01); **indicates a significant difference (*P* < .01) calculated by Student's *t* test

We established gastric cancer xenograft model using BALB/c nude mice. Tumor tissues from the SGC‐7901 cells transducted with sh‐circSMC3 grew dramatically slower than the cells transducted with the control group (mpCDH is a vector that we used to transient transfect or stable transduce shRNA into host cells) (Figure 6A‐C). Furthermore, we measured the RNA levels of miR‐4720‐3p in sh‐circSMC3 tumors. The expression levels of miR‐4720‐3p were increased in sh‐circSMC3 group compared with the control group (Figure 6D). Additionally, we measured the *TJP1* levels in sh‐circSMC3 tumors. *TJP1* levels were decreased in sh‐circSMC3 group (Figure 6E). Together, these results suggested that *TJP1* was the functional protein of circSMC3/miR‐4720‐3p.

## 4 DISCUSSION

Noncoding RNAs, including circRNAs, microRNAs (miRNAs), and long noncoding RNAs are extensively investigated in many cancer types.^17^, ^18^, ^19^ Both long non‐coding RNAs and circRNAs do not encode proteins; circRNAs are characterized by the covalent linkages and lack of the 3′ and 5′ end. In gastric cancer, circRNAs had been found to play a carcinogenic or anticarcinogenic role in tumorigenesis.^20^, ^21^

In the present study, dysregulated circRNA (circSMC3) was evidently upregulated in gastric cancer tissues. Five overexpressed circRNAs (hsa_circ_0025202, hsa_circ_0020123, circSMC3, hsa_circ_0132266, and hsa_circ_0005519) were identified. Another five under‐expressed circRNAs were also identified, including hsa_circ_0079593, hsa_circ_0001785, hsa_circ_0126991, hsa_circ_0020397, and hsa_circ_0002577.

Functional cellular experiments indicated that silencing circSMC3 inhibited the gastric cancer cell proliferation and motility. CircRNAs can target miRNAs by acting as miRNA sponge and binding with the RNA‐binding protein (RBP) to exert their function.^22^ Mechanical investigation indicated that circSMC3 targeted miR‐4720‐3p as a miRNA sponge, which was examined using a luciferase reporter assay and western blotting.

Further exploration indicated that *TJP1* was the target of miR‐4720‐3p, forming the circSMC3/miR‐4720‐3p/*TJP1* axis. The levels of *TJP1* in gastric cancer tissues (stomach adenocarcinoma) are higher than normal tissues based on the GEPIA database.^23^ Although the statistical result is not significant, the increasing *TJP1* in cancer tissues still positively correlate with circSMC3 expression and negatively correlate with miR‐4720‐3p expression.

The role of circRNAs in human cancers had been established in previous studies.^15^, ^17^, ^18^ For example, circRNA hsa_circ_0000199, also termed as circAKT3, was an obviously increased circRNA in cis‐Diaminedichloroplatinum‐resistant patients.^20^ CircAKT3 promoted DNA damage repair and inhibited the gastric cancer cells apoptosis by increasing PIK3R1 expression through sponging for miR‐198.^20^ In addition, circPSMC3 regulates miRNA‐296‐5p/PTEN axis to participate in the progression of gastric cancer.^24^ Further, circPSMC3 serves as a new potential circulating biomarker for detection of gastric cancer.^24^ All these studies suggest that circRNAs target the miRNA as miRNA sponge to modulate the cellular function.

Taken together, our study identified the role of circSMC3 in gastric cancer cells via sponging miR‐4720‐3p to initiate *TJP1* potential. This research characterized the regulation of circSMC3/miR‐4720‐3p/*TJP1* axis and its role in gastric cancer.

## 5 CONCLUSION

Our results provided the first convincing evidence that circSMC3 may be an important oncogene by targeting miR‐4720‐3p in human gastric cancer. However, it might be limited in our research, and further research may be crucial for our future research.

## DISCLOSURE

The authors report no conflict of interest in this work.

## AUTHOR CONTRIBUTIONS

All authors contributed to data analysis, drafting, or revising the article, gave final approval of the version to be published, and agreed to be accountable for all aspects of the work.

## DATA SHARING STATEMENT

The original data are available from the corresponding author upon request.
